# Turkish Adaptation and Validation of Patient Participation Questionnaire (PPQ) ‡

**DOI:** 10.3390/healthcare13040358

**Published:** 2025-02-08

**Authors:** Adil Aydoğdu, Mehmet Yorulmaz

**Affiliations:** 1 Department of Health Management, Institute of Health Sciences, Selcuk University, Selcuklu, 42010 Konya, Türkiye; 2 Department of Health Management, Faculty of Health Sciences, Selcuk University, Selcuklu, 42010 Konya, Türkiye; mehmetyorulmaz@selcuk.edu.tr

**Keywords:** psychometric, reliability and validity, involvement, patient participation

## Abstract

**Background/Objectives**: The concept of patient participation is increasingly recognized as an important component in many areas, such as redesigning healthcare processes, improving patient safety, increasing satisfaction, and managing chronic diseases. In this context, measuring the level of patient participation in healthcare services is an important factor. The “Patient Participation Questionnaire” is a tool used to assess patients’ evaluations of their participation in their in-hospital care. The absence of a scale in the Turkish literature that measures this concept reveals the importance of this research. **Methods**: In this study conducted in a tertiary public hospital in Turkey, the final scale translated into Turkish was applied to 355 people using the convenience sampling method. In addition to the “Patient Participation Scale”, the “Patient Satisfaction Scale” was used for context validity in the study. Data were analyzed with SPSS 27 and AMOS programs. **Results**: As a result of the confirmatory factor analysis, the scale, which originally consisted of 16 questions and four dimensions, was adapted to Turkish as 14 questions and four dimensions. As a result of confirmatory factor analysis, the goodness of fit values of the scale were found to be x^2^/sd = 2.53, GFI = 0.93, AGFI = 0.90, CFI = 0.93, RMSEA = 0.066, RMR = 0.041, and NFI = 0.90. These values are within the acceptable and good fit level ranges. As a result of the correlation analysis performed for context validity, it was determined that there was a positive significant relationship between the adapted patient participation scale and the patient satisfaction scale (r = 0.692, *p* < 0.001). In addition, the internal consistency coefficient of the scale was examined to determine the reliability of the scale, and it was revealed that the scale was reliable at a good level (α = 0.86). **Conclusions**: Based on the findings, it was revealed that the “Patient Participation Scale” developed in English is a valid and reliable measurement tool in Turkish culture.

## 1. Introduction

The health sector has developed in a constantly progressive manner. From the times when basic health services could not be provided, it has reached a period where there are many private health institutions, many health technologies have become a part of service delivery, remote telemedicine methods have developed, health tourism opportunities have increased, and health services can be easily purchased everywhere with e-health applications. This rapid transformation experienced by the healthcare sector, especially in the early 21st century, has also changed the system’s perspective on patients; an evolution has taken place towards a system that accepts patients as individuals, respects their rights and freedoms, and tries to minimize the existing information asymmetry. One of the important concepts in this transformation has been the concept of patient participation.

Important events that have occurred worldwide in recent years in the field of health (such as COVID-19) have once again shown us that health is an important phenomenon and revealed the importance of individual measures and initiatives in protecting and improving the health of individuals. In addition to the steps that individuals take to protect their health, taking action in line with the recommendations of health professionals has played a key role in the effectiveness of health services and the success of the health system. This process has shown us the importance of the concept of patient participation from a different dimension.

Although the concept of patient participation may seem like a new concept, the ideal of patients participating in their own treatment and care emerged in the 1960s simultaneously with movements in society towards the recognition of the autonomy rights of individuals [1]. Individuals were demanding to be more active as patients and did not want to blindly follow the rules and directives of health professionals. Instead, individuals wanted to be able to express their own opinions on their own bodies and health, to have at least enough information and ideas about the steps to be taken, and to be able to make choices when necessary. Healthcare providers, on the other hand, expected individuals to take responsibility for their health. The American Medical Association (1984) [2] declared that patients have the right to self-determination, and the World Health Organization stated that patient participation in care is not only desirable but also a social, economic, and technical necessity [3,4].

The term patient participation has been used in different meanings for many years. Before explaining the term patient participation, the word participation, which we examined, means to be involved and take part, according to the Cambridge Dictionary [5]. Therefore, it is possible to define patient participation as “involving individuals in their own health care decisions” based on the word participation. On the other hand, the concept of patient participation is a set of phenomena that includes “individual or holistic care, realistic plans based on negotiation, a positive outcome for patients, and encouraging patients to be active rather than passive during their hospital stay” [6].

Patient participation is an important concept on its own, but it is also closely related to other important concepts such as patient empowerment and patient safety. Ensuring that patients take an active role in their own health care has been identified as an important factor in efforts to improve health services for patients at national and international levels. It has been stated that patients can play an important role in reducing risks related to patient safety in healthcare processes, such as helping to avoid medication errors and monitoring adverse events [7]. However, involving patients in the care process provided to them should not mean that patients should assume ultimate responsibility for the safety of the care they receive. Patients should not feel that they will receive substandard care if they do not want to or cannot contribute to their own safety [8]. This situation expresses the other aspect of the concept of “patient participation”. Because there are also views that patient participation in treatment processes should be limited to scientific processes only. Studies measuring whether patients really want to participate in the decision-making process have produced conflicting results, and the evidence for a positive outcome for patients who participate in decisions about their medical care is suggestive rather than definitive. Although not all patients want to take control of their medical care, it is still important that their concerns, wishes, and values are included in decisions about their care [3].

As can be seen in the definitions, the concept of “patient participation” will be a subject of research for many years as an important part of health service delivery. However, when the literature on the subject is examined, the number of studies examining the subject of patient participation rather qualitatively and examining the relationship of the subject with different concepts by measuring the level of patient participation is low. This situation makes it important to establish the concept of patient participation on a more solid ground by investigating it in different ways. It is thought that the low number of such studies, especially in the national literature, is due to the lack of many measurement tools for measuring patient participation. Although there are studies in the literature aimed at measuring patient participation in different dimensions and with different types of questions, there is no measurement tool related to the participation of inpatients in the treatment processes. Therefore, there is a need for a measurement tool with tested validity and reliability to be used in measuring the level of patient participation in the national literature. In this context, the study was conducted in order to meet this need and will make a significant contribution to the field in terms of the results obtained.

In line with all of these, the aim of this methodological study is to test the validity and reliability of the Turkish version of the “Patient Participation Questionnaire (PPQ)” scale developed by Berg et al. (2020) [9]. In this context, the research was conducted on adult individuals who received inpatient treatment from a tertiary hospital in Turkey.

## 2. Materials and Methods

### 2.1. Population and Sample

The population of the research consists of adult individuals who received inpatient treatment services from Selçuk University Faculty of Medicine Hospital in Konya, a metropolitan city located in the Central Anatolia Region of Turkey. Selçuk University Faculty of Medicine Hospital started accepting patients in 2009 and is a tertiary hospital with a bed capacity of 990, serving 1,094,278 people living in Selçuklu, Meram, and Karatay, the central districts of Konya [10], and surrounding provinces and districts.

In scale development and adaptation studies, it is recommended that the required sample size be 5–10 times the number of items [11,12,13]. According to Kline (2005), in order to test the model in factor analysis and to provide sufficient degrees of freedom, a sample size of 20 times the number of items should be reached [14]. In this context, the targeted sample size (16 × 20) was determined as 320, the target was to reach 350 people, and 358 people were reached. During the data control process, 3 questionnaire forms were excluded from the evaluation because they did not meet the research criteria. As a result, the sample of the study consisted of 355 adult individuals who received inpatient treatment from Selçuk University Medical Faculty Hospital.

#### Demographic Characteristics of Participants

Table 1 shows the demographic information of the participants in the study. As seen in Table 1, 50.7% of the participants in the study were female, 20% were 66 years or above; 36.6% were primary school graduates; 53.5% were unemployed, self-employed, or students. According to income level, it is seen that 61.7% of the participants have income below the minimum wage.

### 2.2. Data Collection Tools

The survey form used in the collection of research data consists of 3 sections: personal information form, “Patient Participation Questionnaire” and “Patient Satisfaction Scale”.

Personal Information Form: The first section of the survey form, the personal information form, contains 5 questions that provide information about the sample in general, including “age, gender, education status, occupation, and income status”.

Patient Satisfaction Scale: The patient satisfaction scale used in the study was developed by Hawthorne et al. (2006) [15] and adapted to Turkish by Temeloğlu Şen and Berk (2022) [16]. The scale, consisting of 7 items and a single dimension, was created in a 5-point Likert style. As the scores approach 5, the satisfaction level increases. The general Cronbach alpha value of the scale was found to be 0.87 in the development study.

Patient Participation Questionnaire: The PPQ, which was adapted into Turkish within the scope of the study, was developed by Berg et al. (2020) [9]. The scale consisted of 16 questions and 4 dimensions in the original study. In this study, it was adapted to Turkish culture with 14 questions and 4 dimensions as a result of confirmatory factor analysis. The dimensions of the scale include a participation/support dimension (3 items), an information dimension (3 items), a communication/cooperation dimension (3 items), and a patient/healthcare personnel relationship (5 items). The scale was prepared in a 4-point Likert type as 1—Not at all; 2—Very little extent; 3—To a certain extent; and 4—To a great extent. The scale has no cut-off point. The scores obtained from the scale approaching 4 indicate a high level of patient participation, while those approaching 1 indicate a low level of patient participation. In addition, the Cronbach alpha value of the scale was found to be 0.86 in this study.

#### Translation and Cultural Adaptation of the PPQ

The adaptation stages of the scale were carried out by using the literature [17,18,19]. The first of the stages was the establishment of the translation committee. PPQ developed by Berg et al. (2020) [9] was first translated into Turkish separately by five academics who know English and Turkish well. The translations obtained were evaluated by two different people who had a good command of English and Turkish and converted into a single Turkish form in the most appropriate way.

The Turkish form was then translated into English by two people whose native language is English and who have a good command of Turkish and who were not involved in the other stages of the translation. The resulting form was compared with the original version of the scale, and a re-evaluation was made. As a result of this comparison, the translated version of the scale was found to be compatible with the original scale.

In the last case, the scale items translated into Turkish were evaluated by eleven experts in the field of health as “appropriate”, “appropriate but needs correction”, and “not appropriate” and the obtained data were organized, and the content validity index was calculated. Lawshe’s method was used to calculate the CVI [20]. As a result of the analysis, it was seen that the CVI, which was 0.94 for the general scale, was within the acceptable value range of over 0.80 [13,21].

The data obtained after expert opinions were evaluated by the research team, and the scale was finalized after the necessary adjustments. Before the survey form was finalized, it was converted into an online survey form and sent to a group of 30 people via Google Forms to evaluate whether there were any unclear statements, and as a result of the feedback, the final Turkish form of the scale was created.

After the final form of the scale was applied to the sample group using a face-to-face survey technique, the obtained data were transferred to the computer environment. In the next stage, the construct validity of the scale was tested. In this context, confirmatory factor analysis of the scale was performed using the AMOS 22 program. In order to test the context validity, a correlation analysis was performed between the “Patient Participation Questionaire” adapted to Turkish and the “Patient Satisfaction Scale” using the SPSS 27 program. In addition to all these, the Cronbach alpha values of the scale and its dimensions were examined using the SPSS 27 program within the scope of the reliability of the “Patient Participation Scale” adapted to Turkish. The findings in question are given in the next section.

### 2.3. Data Collection Process

The first phase of the research started with the translation phase on 2 May 2024, and the final survey form to be applied was finally created on 9 July 2024. After the final survey form was created, the implementation process of the scale started on 10 July 2024 and was stopped on 18 July 2024 due to the sufficient number.

During the collection of research data, before moving on to the survey form, participants were given face-to-face information about the “purpose of the study and the researchers”, and participants who wanted to participate in the research voluntarily were asked to fill out the survey form. Since the research questions required individuals to have received inpatient treatment at the hospital for at least 1 day (for reasons such as surgery, observation, psychological or physical treatment, etc.), participants were asked whether they met these criteria before asking the research questions. Individuals who had not received inpatient treatment from the hospital where the research was conducted within the last year were not included in the study. The survey forms were answered personally by individuals who had no obstacles to reading or answering the survey. Data were collected by participants who had any obstacles to answering the questions (such as age-related disabilities, physical disabilities (broken arm, etc.), or illiteracy) by having the survey questions read to them in an official language by the researchers. The research data were collected between 10 July 2024 and 18 July 2024 by face-to-face survey technique. During the collection of research data, the participants were informed about the study before proceeding to the questionnaire form, and the participants who voluntarily wanted to participate in the study were asked to fill out the questionnaire form.

### 2.4. Limitations and Assumptions

Although this study provides evidence that the PPQ is a valid and reliable instrument to measure Turkish inpatients’ participation in the treatment process, it also has some limitations. The study was conducted only on patients who received inpatient care in a tertiary public hospital. In addition, the fact that the scale data were collected face-to-face ensured that the study was limited to the hospital environment. Patients were not segregated by outpatient clinic, and data were collected overall. It is assumed that participants understood the questions correctly and answered them objectively. In addition, there are no other psychometric studies to discuss this measurement tool. Consequently, its psychometric properties should be examined in a broader validation context in the future.

### 2.5. Data Analysis

While the AMOS 22 program was used for confirmatory factor analysis in the data analysis phase, Microsoft Office Excel and SPSS 27 programs were used for data organization, descriptive analyses (frequency, percentage average), Cronbach alpha, and correlation analyses.

In the validity phase of the scale, firstly, language and scope validity, and then structure and context validity, were performed. In the reliability phase, Cronbach alpha values expressing the internal consistency coefficient were examined.

### 2.6. Ethical Considerations

Before starting the study, ethical permission was obtained from the Selcuk University Faculty of Health Sciences Non-Interventional Research Ethics Committee. After the approval of the Ethics Committee, approval was obtained from the Selcuk University Faculty of Medicine Hospital, where the study would be conducted. Participation in the study was voluntary, and all participants gave their informed consent before data collection began.

## 3. Results

In this section, the standard coefficient values of the path diagram before and after the first- and second-level confirmatory factor analysis of patient participation are given. In addition, a correlation analysis between the patient participation scale and the patient satisfaction scale is included for context validity.

### 3.1. Validity Analysis

**Figure 1:**
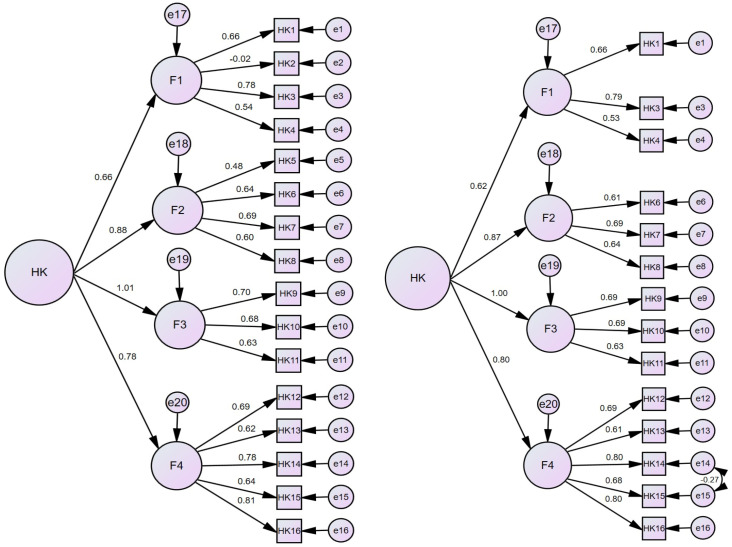
Before and after second-level CFA path diagram of PPQ.

Figure 1 shows the second-level confirmatory factor analysis (CFA) results (path diagram) of the PPQ.

The first path diagram seen in the graphs given as Figure 1 belongs to the first stage of the analysis, and it was decided what to do to improve the goodness of fit values. In this context, in order to correct the goodness of fit values, it was decided to remove the 2nd scale expression (To what extent did you allow the health personnel to make decisions on your behalf about your treatment process?) whose standardized item loadings were below 0.32 and the 5th scale expression (To what extent did the health personnel provide you with special information other than routine information?) which caused a contradiction between the scale expressions and the standardized item loading [22]. Finally, in order to improve the goodness of fit values of the model, a covariance connection was made between the 14th and 15th items of the scale [23,24,25].

Table 2 includes the first- and second-level CFA results of the PPQ and information on good fit and acceptable fit values in the literature. According to Table 2, it was determined that the CFA findings of the PPQ showed acceptable and good fit according to the values stated in the literature [12,23,24,25,26,27,28].

Table 3 shows the significance levels of the items forming the PPQ dimensions. According to the findings, it was determined that the dimensions forming the patient participation scale dimensions and the factor loadings of the 14 items forming the dimensions showed a significant distribution (*p* < 0.001). In the next stage, the factors and items obtained were examined and named by the research team. In naming the factors, in addition to the scale items, Berg et al. (2020) [9], who developed the scale, was used to ensure that the original factors of the scale were adhered to.

### 3.2. Internal Validity

Table 4 shows the factor names, scale items, and total correlation analysis results for the PPQ. The Cronbach alpha value is used to measure the internal consistency of the scales. The Cronbach alpha value determines whether the scale items have a homogeneous structure [29]. The Cronbach alpha value used in the Likert-structured scales indicates reliability when it is between 0.60 and 0.79 and high reliability when it is between 0.80 and 1.00 [19,30,31,32].

According to the findings, the Cronbach alpha value of the PPQ was calculated as 0.87. This information shows that the scale has high reliability. In addition, when the values of the scale dimensions are examined, similarly reliable and highly reliable results are seen.

In addition, the patient satisfaction scale was used in the study in order to test the context validity, and the results of the scales are given in Table 5. The table also includes the correlation analysis between the total score of the PPQ and its sub-dimensions and the correlation analysis results between the PPQ and the Patient Satisfaction Scale (PSS), which were conducted to test the context validity.

According to the findings, all of the relationships between the patient participation scale and its sub-dimensions were found to be statistically significant and positive, and the correlation coefficients varied between 0.69 and 0.84 (*p* < 0.001). In addition, when the Pearson correlation test results between the PPQ and PSS were examined within the scope of context validity, a positive significant relationship was determined between the PPQ and the PSS (r = 0.69; *p* < 0.001). As patient participation increases, patient satisfaction also increases.

The subject of patient satisfaction is one of the important issues in the field of health management, and there are various studies in the literature on the factors affecting patient satisfaction. However, when evaluated in general, Kavuncubaşı and Yıldırım (2012) examined the factors affecting patient satisfaction in three categories: related to the patient and their relatives; related to the service providers; and physical environmental or institutional factors [33]. In another study, Esatoğlu (1997) stated that patient satisfaction consists of the patient–doctor relationship, patient–nurse relationship, patient–other hospital personnel relationship, information, nutrition services, physical and environmental conditions, bureaucratic procedures, trust, and wage dimensions [34]. As can be seen, although the dimensions of patient satisfaction and the factors affecting satisfaction are not different from the dimensions that constitute the PPQ, many factors examined within the scope of patient participation directly or indirectly affect patient satisfaction. In addition to the findings given in Table 5, the means of the scale dimensions were examined, and it was seen that the skewness and kurtosis values were between “−1 and +1”, and these results obtained show that the data did not deviate from the normal distribution [31].

## 4. Discussion

In recent years, companies in many sectors have made customers a part of the production process, thus minimizing the risks that may arise in the production process, creating customer satisfaction, and establishing customer loyalty, thus ensuring the customer potential in the future. This situation also manifests itself in the healthcare sector as “Patient Participation”. Although the concept of patient participation is used in different ways, this concept generally aims to ensure that individuals participate in the treatment process during the provision of healthcare services, their opinions are taken into consideration, their thoughts are given importance, the patient takes an active role in the treatment process, and the care recommendations to be given are meticulously implemented by the patient.

Ensuring patient participation provides various benefits for individuals, as well as institutional and social benefits such as the success of the treatment process and the prevention of unnecessary use of health services. These and similar factors make it important to measure and reveal the level of patient participation. There are various studies on the subject in the national literature, but there is no measurement tool that has been conducted on patients who have received inpatient treatment. In addition, when existing measurement tools were examined, a measurement tool that measures the level of patient participation in a short format similar to the scale adapted within the scope of the study could not be found. Based on this, the validity and reliability of the new version of the PPQ were evaluated in the study based on data obtained from patients who received inpatient treatment from a university hospital. In patient participation studies, since national and international data that can be compared with different factors can be obtained by using a valid and reliable tool, the Turkish adaptation of the scale is of great importance.

The study findings showed good internal consistency, content validity, and construct validity, and the results obtained showed that the patient participation scale can measure the participation levels of individuals who received inpatient treatment services from Turkish hospitals. In addition, the items in the scale contributed to the expected sub-factor and provided evidence for construct validity. In order to adapt the patient participation questionnaire, which was originally developed as a 16-item and 4-dimensional version, to Turkish culture, confirmatory factor analysis was conducted after language and scope adaptation. Based on the modification indices in the analysis, the 2nd item in the original scale (To what extent did you allow the healthcare personnel to make decisions on your behalf about your treatment process?) and the 5th item (To what extent did the healthcare personnel provide you with special information other than routine information?) that caused a contradiction in the scale expressions and standardized item loading were removed. The model was re-run with 14 items and four dimensions by establishing a covariance relationship between the 14th and 15th items. As a result of the analysis, it was determined that the goodness of fit values of the model belonging to the PPQ showed good and acceptable fit [12,23,24,25,26,27,28]. In order to test the context validity, the correlation between the PPQ, which was adapted into Turkish, and the patient satisfaction scale was examined, and a significant positive relationship was found between the two scales (r = 0.69). In addition, the internal consistency coefficient of the scale (Cronbach’s alpha) also showed that the scale was an acceptably reliable measurement tool (α = 0.86) [19,30,31,32]. There are currently two scales in the Turkish literature regarding the concept of patient participation. However, the 16-item patient participation scale adapted in this study has several differences from other scales. For example, the “Patient Health Engagement Scale (PHE-s)”, the validity and reliability of which was performed by Usta et al. in 2019, aimed to evaluate the emotional, behavioral, and cognitive competencies of individuals with chronic diseases during the care of patients. The scale consists of five items and was studied on 114 patients, and the evaluation scale consists of a structure with seven options among the four statements under each question, and a Likert-style structure was not used. The ordinal alpha value of the scale was found to be 0.80 [35]. “Participation in Patient Care Scale”, which was adapted into Turkish by Bilgin et al. in 2024, was conducted on 214 patients and consists of 21 items. The Cronbach’s alpha value was calculated as 0.92 on the 5-point Likert-type scale [36]. In the international literature, in different adaptation studies (China and Denmark) where patient participation was addressed in various dimensions, Cronbach’s alpha coefficient was calculated as 0.91 and 0.89, respectively; further cross-cultural translation and adaptation into other languages may also be important [9,37].

## 5. Conclusions

As a result, the patient participation questionnaire developed by Berg et al. (2020) [9] has been shown to be a valid and reliable measurement tool in Turkish society. In addition, this scale, which was adapted into Turkish, was applied to individuals who received inpatient treatment services in a specific hospital; thus, its validity and reliability were proven on this group. Despite the patient participation scales that generally exist in the national literature, since there is no measurement tool that can be used to measure the level of inpatient participation, it is thought that this scale, whose validity and reliability were performed in Turkish, will make an important contribution to filling the gap in the literature and to responding to the lack of measurement tools on patient participation. In future studies in the field of patient participation, the patient participation level issue can be studied alone, as well as its effects on various factors, or the effects of various factors on the level of patient participation can be investigated.

On the other hand, it is recommended that the scale that was added to the literature as a result of the study be applied to patients receiving inpatient treatment in different hospital types (e.g., private hospitals) in future studies, comparisons be made between different hospitals, and outpatient clinic-based studies be conducted. Another limitation could be that the study was conducted in a public hospital, which may have caused patients to refrain from expressing too many negative opinions about their participation in the treatment process. In addition, since the research data being limited to the hospital environment alone will cause individuals who are not in the hospital environment but meet the research criteria not to be reached, it is also recommended that the research be conducted online. In addition, the patient satisfaction scale was used in the study in order to test the validity of the context, and the results showed that the scale can be used with different scales. Therefore, it is recommended that the patient participation scale be used with scales related to different factors that may affect individuals during the treatment process (satisfaction, hospital preference, etc., intention to recommend, patient trust, etc.). In addition, it is predicted that various results can be revealed when used with scales in the literature regarding patient–physician and patient–healthcare professional communication, and it is presented as a suggestion for further studies.

## Figures and Tables

**Table 1:** Demographic features of the participants.

Demographic Features	Choices	N	%
Gender	Female	180	50.7
	Male	175	49.3
Age	18–25 years	54	15.2
	26–35 years	40	11.3
	36–45 years	59	16.6
	46–55 years	70	19.7
	56–65 years	61	17.2
	66 years or above	71	20.0
Educational Status	Illiterate	39	11.0
	Primary School	130	36.6
	Secondary School	58	16.3
	High School	69	19.4
	University	47	13.2
	Not specified	12	3.4
Occupation	Public	32	9.0
	Private Sector	47	13.2
	Retired	74	20.8
	Unemployed/Student/Freelance	190	53.5
	Not specified	12	3.4
Income Status	Below Minimum Wage	219	61.7
	Minimum wage	71	20.0
	Between 2 and 3 times the minimum wage	37	10.4
	3 times the minimum wage and more	10	2.8
	Not specified	18	5.1
Total		355	100.00

**Table 2:** Goodness of fit indices used in first- and second-level CFA of PPQ.

Fit İndices	Good Fit	Acceptable Fit	First-Level Model	Second-Level Model
x^2^/Sd	0 ≤ x^2^/sd <2	2 ≤ x^2^/sd ≤ 3	2.354	2.533
GFI	0.95 ≤ GFI ≤ 1.00	0.90 ≤ GFI ≤ 0.95	0.940	0.932
AGFI	0.90 ≤ AGFI ≤ 1.00	0.85 ≤ AGFI ≤ 0.90	0.910	0.901
CFI	0.90 ≤ CFI ≤ 1.00	0.85 ≤ CFI ≤ 0.90	0.946	0.936
NFI	0.95 ≤ NFI ≤ 1.00	0.90 ≤ NFI ≤ 0.95	0.910	0.900
RMSEA	0 ≤ RMSEA ≤ 0.05	0.05 ≤ RMSEA ≤ 0.08	0.062	0.066
RMR	<0.05	<0.08	0.036	0.041

GFI: goodness of fit index, AGFI: adjusted goodness of fit index, CFI: comparative fit index, NFI: normed fit index, RMSEA: root mean error of approximation, RMR: root mean residual.

**Table 3:** Significance levels of the statements constituting the PPQ dimensions.

			Estimate	Standardized Estimate	S.E.	C.R.	*p*
1	Factor 1	Item 1	1	0.66			
2		Item 3	1.11	0.77	0.12	9.57	***
3		Item 4	1.04	0.56	0.13	8.26	***
4	Factor 2	Item 6	1	0.61			***
5		Item 7	1.10	0.68	0.12	9.41	***
6		Item 8	1.06	0.65	0.12	9.19	***
7	Factor 3	Item 9	1	0.69			***
8		Item 10	1.08	0.69	0.10	11.17	***
9		Item 11	0.89	0.64	0.09	10.47	***
10	Factor 4	Item 12	1	0.70			
11		Item 13	0.89	0.60	0.09	10.40	***
12		Item 14	1.27	0.80	0.10	13.28	***
13		Item 15	1.26	0.68	0.11	11.32	***
14		Item 16	1.27	0.80	0.09	13.55	***

***: *p* < 0.001.

**Table 4:** Item correlation analysis and reliability values of the PPQ.

Factors	Items		Corrected Total Question Correlation	When Question is Deleted	Factors	Total Cronbach Alpha
Participation/Support Dimension	1	To what extent did you have the opportunity to be involved in decisions regarding your treatment?	0.44	0.87	0.68	0.87
	2	To what extent did you work with healthcare personnel on issues related to your treatment?	0.52	0.86		
	3	To what extent were your needs met by the nurses so that you could continue your daily routines?	0.34	0.88		
Information Acquisition Dimension	4	To what extent were you informed about your treatment during your hospital stay?	0.53	0.86	0.68	
	5	To what extent did the information provided by healthcare personnel help you understand your health condition and treatment?	0.56	0.86		
	6	To what extent was the information you received understandable?	0.53	0.86		
Communication/Collaboration Dimension	7	To what extent did you have the opportunity to discuss your treatment with healthcare personnel?	0.63	0.86	0.71	
	8	To what extent did healthcare personnel value your views and experiences regarding your health condition?	0.61	0.86		
	9	To what extent did you experience that the individual information you shared with healthcare personnel was shared among the healthcare personnel involved in your treatment?	0.58	0.86		
Patient/ Healthcare Personnel Relationship	10	To what extent did you trust the professional expertise of healthcare personnel?	0.53	0.86	0.83	
	11	To what extent did healthcare personnel value your individual views and needs during your treatment?	0.59	0.86		
	12	To what extent did healthcare personnel treat you sincerely?	0.58	0.86		
	13	To what extent did you have the opportunity to share your thoughts and concerns with healthcare personnel?	0.52	0.86		
	14	To what extent did you feel that your relationship with healthcare personnel was based on mutual respect?	0.63	0.85		

**Table 5:** Context validity of the PPQ and its dimensions.

Scale and Dimesions	Mean ± SD		1	2	3	4	5	6
1. PATIENT PARTICIPATION	3.05 ± 0.48	r	1					
2. Participation/Support Dimension	2.64 ± 0.78	r	0.69 **	1				
3. Information Acquisition Dimension	3.16 ± 0.55	r	0.78 **	0.41 **	1			
4. Communication/Collaboration Dimension	2.96 ± 0.58	r	0.84 **	0.49 **	0.58 **	1		
5. Patient/Healthcare Personnel Relationship	3.28 ± 0.58	r	0.83 **	0.29 **	0.58 **	0.65 **	1	
6. PATIENT SATISFACTION	19.81 ± 4.04	r	0.69 **	0.46 **	0.53 **	0.52 **	0.63 **	1

N = 365; ** *p* < 0.001.

## Data Availability

The database used in this work is available upon request to the corresponding author.
